# Discovery, phylogenetic, and comparative genomic analysis of novel avian gammacoronaviruses identified in feral pigeons (*Columba livia domestica*)

**DOI:** 10.1128/jvi.01112-25

**Published:** 2025-08-20

**Authors:** Mohamed E. El Zowalaty, Louis J. Taylor, Yongwoo Son, Heesu Lee, Adam M. Rubrum, Richard J. Webby, Stephen A. Bustin, Sean G. Young, Sun-Hak Lee, Dong-Hun Lee, Matthew B. Frieman

**Affiliations:** 1 Division of Virology, Department of Infectious Diseases, St. Jude Children's Research Hospital 5417, Memphis, Tennessee, USA; 2 Center for Pathogen Research, Department of Microbiology and Immunology, University of Maryland School of Medicine 200790, Baltimore, Maryland, USA; 3 Wildlife Health Laboratory, College of Veterinary Medicine, Konkuk University 34965, Seoul, Republic of Korea; 4 Avian Disease Laboratory, College of Veterinary Medicine, Konkuk University 34965, Seoul, Republic of Korea; 5 Molecular Diagnostics Unit, Medical Technology Research Centre, Anglia Ruskin University 2369, Chelmsford, United Kingdom; 6 Peter O’Donnell Jr. School of Public Health, The University of Texas Southwestern Medical Center 12334, Dallas, Texas, USA; Emory University School of Medicine, Atlanta, Georgia, USA

**Keywords:** coronavirus, avian, gammacoronaviruses, feral pigeons, *Columba livia domestica*, genome sequence, next-generation sequencing, pan-coronavirus, evolution

## Abstract

The COVID-19 pandemic caused by SARS-CoV-2 has intensified efforts to identify emerging coronaviruses (CoVs) across diverse hosts. Spillover events, where CoVs transition from wildlife reservoirs to other species, can lead to infections with varying clinical outcomes, emphasizing the need for continued surveillance. Understanding the diversity and distribution of both known and novel CoVs in wildlife reservoirs is crucial for predicting and preventing future spillover events. In this study, we employed Illumina’s Pan-CoV library panel and next-generation sequencing (NGS) to identify five novel avian-associated gammacoronavirus genomes from cloacal and oropharyngeal swabs of clinically healthy feral pigeons captured in Durban, KwaZulu-Natal Province, South Africa, in 2018. The genome sequences, ranging from 27,582 to 27,611 nucleotides, clustered with gammacoronaviruses in phylogenetic analyses but formed a distinct clade. Comparative analysis of the five conserved domains in the ORF1ab coding sequence with other gammacoronaviruses revealed Pairwise Patristic Distances (PPD) that exceeded both species and subgenus demarcation cutoffs. These findings highlight the critical need for ongoing surveillance to enhance understanding of CoV diversity, host range, and potential for cross-species transmission, aligning with One Health principles. Based on these results, we propose *Gammacoronavirus columbae* as a new species for these pigeon gammacoronaviruses that clusters with the recently reported duck gammacoronaviruses to form a novel subgenus within Gammacoronavirus.

**IMPORTANCE:**

Although coronaviruses are significant pathogens affecting a wide range of hosts, surveillance efforts have predominantly focused on wild mammals, leaving the diversity of avian coronaviruses largely underexplored. Here, we report the detection of novel gammacoronaviruses from feral pigeons in South Africa and propose revisions to the current taxonomic classification of Gammacoronavirus based on genetic distance analyses. These findings highlight the potential role of wild birds in the dissemination of novel coronaviruses, analogous to their established involvement in the transmission of avian influenza viruses. Our study also highlights the utility of next-generation sequencing (NGS) technologies in uncovering the hidden diversity of viruses in wildlife populations. Finally, this study reinforces the need for ongoing surveillance, continued vigilance, and further research into avian coronaviruses. The ongoing highly pathogenic avian influenza (HPAI) outbreaks in the USA have demonstrated the devastating impact of emerging avian viruses on wildlife, agriculture, and public health. Given the unpredictability of coronavirus evolution, failing to monitor their diversity and potential for cross-species transmission risks leaving us unprepared for future outbreaks. This study reinforces the urgent need for proactive, large-scale genomic surveillance of wildlife reservoirs to identify emerging CoVs before they become significant threats to animal and human populations.

## INTRODUCTION

Coronaviruses (CoVs) are among the largest known eukaryotic enveloped RNA viruses, possessing a positive-sense, single-stranded, linear, nonsegmented RNA genome of approximately 30 kilobases in length (1). They are characterized by high genetic diversity due to frequent mutations and recombination events (2), resulting in the appearance of different genotypes and distinct viral lineages, which can lead to the emergence of new variant viruses (3, 4). This variability facilitates host adaptation and interspecies transmission, increasing their zoonotic potential (5, 6)

Coronaviruses circulate continuously among all vertebrates including birds, posing an ongoing threat to potential hosts such as livestock, companion animals, and humans. Avian coronaviruses belong to the order *Nidovirales*, family *Coronaviridae*, subfamily *Orthocoronavirinae*, genus *Gammacoronavirus*, subgenus *Igacovirus*, and are classified within the genera gamma- and delta-CoVs. These viruses infect both domesticated and wild birds, causing highly contagious and economically significant diseases in avian species and poultry worldwide (7, 8). The avian infectious bronchitis virus (IBV), the prototype gamma CoVs, infects domestic fowl (*Gallus gallus* known as the chicken) and causes infectious bronchitis disease (9), a notifiable condition according to the World Organization for Animal Health (WOAH).

Gammacoronaviruses can cause respiratory, hepatic, enteric, and neurological disease (10) although many infections occur without apparent clinical signs (9). The detection of avian CoVs other than IBV was reported in wild birds in many parts of the world including North America, South America, Europe, Australia, and Asia (11–17), but not Antarctica (18). Avian CoVs were also discovered in healthy wild birds, highlighting their significant role in the epidemiology and evolutionary role of other gamma- and delta CoVs (19).

Coronaviruses have been detected and characterized in migratory and non-migratory wild birds including birds belonging to the family *Columbidae* (Order *Columbiformes*) (20). *Columbidae* is a family consisting of more than 300 species of pigeons and doves occurring worldwide (21). Pigeons are ubiquitous, synanthropic, and highly mobile potential asymptomatic vectors capable of free flying which allow moving and spreading of viruses over large distances and across different geographical borders (11, 22). Wild birds have been implicated in the spread of highly pathogenic H5Nx avian influenza A viruses (23, 24). Feral pigeons are one of the most globally invasive avian species and are a reservoir of numerous pathogens (22) posing continuous threats to veterinary and public health (25). The close proximity of pigeons to other domestic and wild animals, as well as to humans (26), could facilitate evolution and recombination events such as in case of coronaviruses potentially leading to new viral variants with varying pathogenicity and tropism (27). Although coronaviruses in pigeons have been detected globally, including isolates from Norway, Poland, Spain, and China (4, 6, 28–31), most previous studies utilized short RdRp amplicons (200–400 bp) that provide insufficient resolution for full-genome diversity analysis, and assay sensitivities vary widely between different PCR protocols (32). Consequently, complete genome sequences of avian CoVs, particularly gammacoronaviruses, remain limited, with notably sparse data from wild bird populations in South Africa.

To address this knowledge gap and characterize the genetic diversity of avian-associated gammacoronaviruses, we performed genomic surveillance of feral pigeons in South Africa, an under-sampled host population and geographic region. We identified five novel gammacoronaviruses that substantially expand the known genetic diversity within this viral genus.

## MATERIALS AND METHODS

### Bird capture

**Fig 1:**
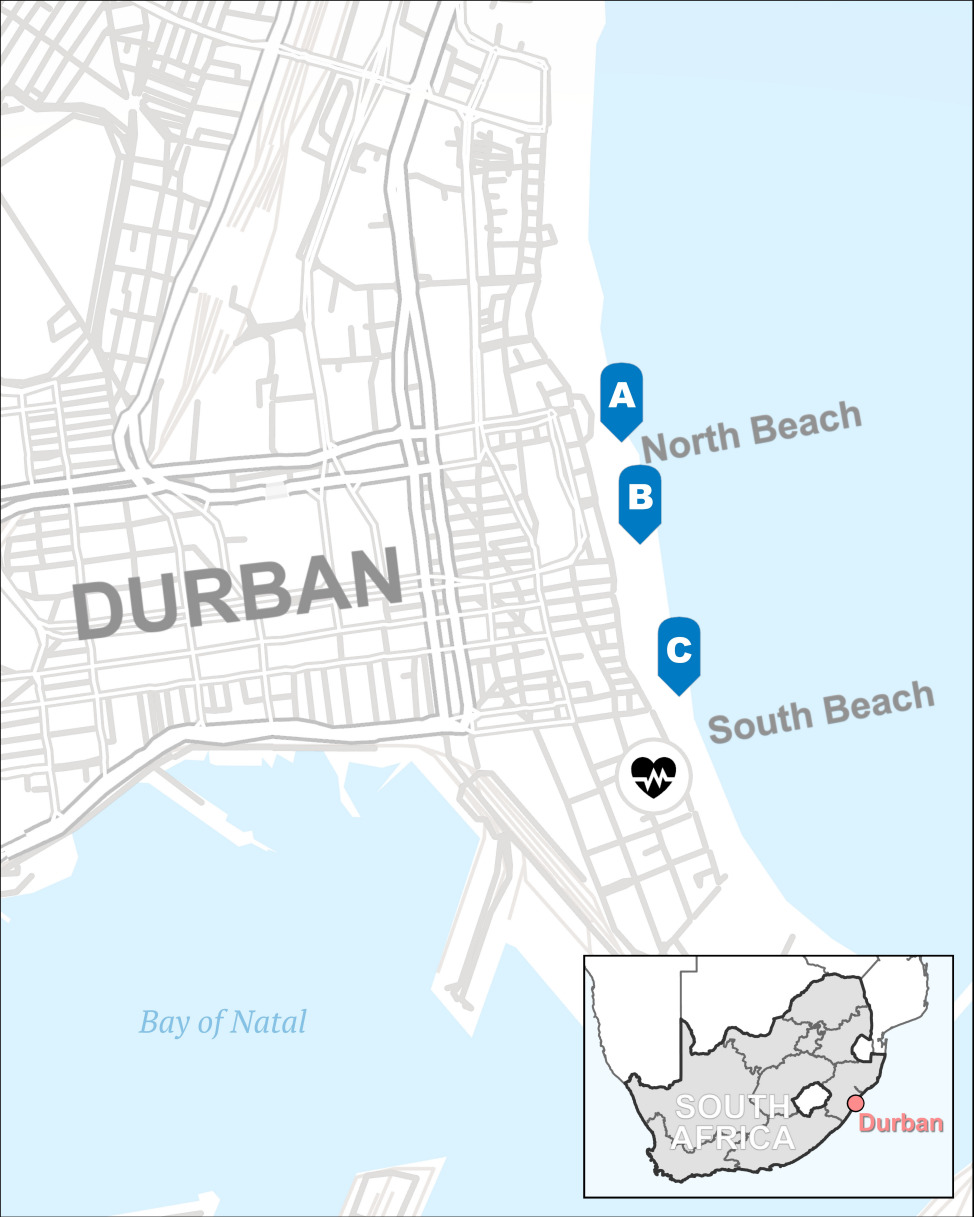
Geographic map showing the locations where the samples in the present study were collected from feral pigeons in Durban, KwaZulu-Natal Province in South Africa. The map was generated using the software ArcGIS Pro (v3.1, Environmental Systems Research Institute [ESRI], Redlands, CA, USA).

Feral pigeons (*Columba livia var. domestica*) (*n* = 81 birds), clinically healthy at the time of sampling, were captured at different locations in KwaZulu-Natal Province in South Africa between February and December, 2018. Pigeon sampling locations in the Greater Durban area are shown in Fig. 1. Feral pigeons roosted in large aggregations in several locations in the Greater Durban area and in KwaZulu-Natal Province, where hundreds to thousands of pigeons often clustered together. Samples were collected individually from each bird, and the birds were immediately released to the wild.

### Virologic sampling

A total of 162 samples including cloacal (*n* = 81) and oropharyngeal (*n* = 81) swab samples were collected during a surveillance project for avian influenza viruses and zoonotic pathogens in South Africa as previously reported (33) using sterile polypropylene swabs with cotton tips (Puritan Medical Products Company, ME, USA) that were immediately placed into 2 mL of viral transport medium containing brain heart infusion broth (Sigma-Aldrich, MO, USA) supplemented with antibiotics (streptomycin [10,000 µg/mL], penicillin [10,000 units/mL], amphotericin B [50,000 µg/mL], and gentamicin [5,000 µg/mL]) as previously described (33). Samples were stored at −80°C, and the cryovials were placed into a cryogenic container and transported to St. Jude Children’s Research Hospital, Memphis, TN, USA, under cold chain for further analysis.

### Viral RNA extraction and quality assessment

Total RNA was extracted from each sample as previously reported (33) using Qiagen RNeasy Mini Kits (Qiagen, Valencia, CA, USA) according to the manufacturer’s instructions. RNA samples were kept frozen at −80°C for further analysis. They were quantified using a fluorimetric RiboGreen assay, and RNA integrity was assessed using capillary electrophoresis (Agilent BioAnalyzer 2100), generating an RNA Integrity Number (RIN). Samples that passed the initial QC step (sample quantity more than 200 ng with an RIN value of 7 or greater) were selected for further screening including Pan-CoV and Illumina sequencing library preparation.

### Influenza A virus RT-qPCR and Newcastle disease virus RT-PCR

RNA samples were tested for influenza A virus (IAV) using real-time reverse transcription-PCR (RT-qPCR) and for Newcastle disease virus (NDV) using conventional RT-PCR as previously reported (33).

### Virus isolation

Virus isolation was performed using two serial passages in specific-pathogen-free (SPF) embryonated chicken eggs using protocols prescribed by the World Organization for Animal Health (OIE) for the most closely related avian pathogens (IBV, influenza viruses, and NDV) as previously reported (33).

### Pan-CoV PCR assay

RNA samples were screened using pan-CoV PCR as previously described by Holbrook et al. (34). The cDNA was synthesized using the RevertAid First Strand cDNA K1622 kit (ThermoFischer, Rockville, MD, USA) according to the manufacturer’s instructions. The PCRs were carried out in 25 µL using 2 µL of cDNA, AllTaq Master Mix 2 (QIAGEN), 2.5 µL CoraLoad (QIAGEN), with each of the primers at 0.4 µM final concentration. Thermal cycling for the first round of PCR was performed at 94°C for 3 min for initial denaturation, followed by 25 cycles of 94°C for 30 s, 48°C for 30 s, 72°C for 1 min, and a final extension at 72°C for 5 min. The second amplification was performed in 25 µL using semi-nested primers at a final concentration of 0.4 µM with μL of PCR product with TopTaq Master Mix 2 (QIAGEN) and 2.5 µL CoraLoad (QIAGEN). Thermal cycling for the second round of PCR was performed at 94°C for 3 min, followed by 40 cycles of 94°C for 30 s, 58°C for 30 s, and 72°C for 1 min. PCR products were visualized using 2% agarose gel electrophoresis. Water controls were used as negative controls in every PCR run and SARS-CoV-2 RNA was used as positive control.

### Whole-genome sequencing of avian CoVs

Avian CoV genomes were amplified and sequenced using the RNA extracted from the original cloacal and oropharyngeal swab specimens as template. Samples were subjected to genome sequencing using the Pan-Coronavirus Panel (Illumina Inc., San Diego, CA, USA) according to manufacturer’s guidelines. Library preparation and sequencing were performed by the University of Maryland Institute of Genome Sciences (Baltimore, MD, USA). Sequencing libraries were prepared using the Pan-Coronavirus Panel capture probe-based sequencing preparation kit (Illumina Inc., San Diego, CA, USA). Sequencing was performed on the Illumina MiSeq instrument (Illumina Inc., San Diego, CA, USA) using the v2 reagent kit (2 × 250 nt paired-end chemistry).

### Bioinformatic and CoV genome sequence analyses

Draft genome assembly was carried out using the Sunbeam pipeline (35) and cross-checked using the DRAGEN Microbial Enrichment workflow (Illumina Inc., CA, USA). The Sunbeam pipeline carries out read trimming and quality filtering using Cutadapt and Trimmomatic (36, 37) followed by assembly using MEGAHIT (38), identification of contigs with CoV homology by BLAST (39). Contigs with CoV homology were subjected to a second round of assembly using CAP3 (40) using the *sbx_select_contigs* extension (https://github.com/ArwaAbbas/sbx_select_contigs) (41, 42) and then scaffolded into draft genomes in samples where CoV sequence was recovered. To obtain more intact genomes, the pigeon gammacoronavirus sequences were further assembled using SPAdes v4.0.0 (43). The resulting draft genome sequences were polished by aligning reads to the assembled genomes using Minimap2 v2.2.0 (44), with sequence visualization and analysis performed using Geneious Prime 2025.1.3. Final viral genome sequences were deposited in the GenBank database at National Center for Biotechnology Information (NCBI) under the accession numbers OR639835, OR810020, OR909854, PP236386, and PV863150.

### Comparative genomic analyses of novel gamma CoVs

 Pigeon gammacoronavirus genome sequences reported in this study were annotated using Geneious Prime 2025.1.3 with NCBI reference gammacoronavirus sequences. For species demarcation, publicly available 920 gammacoronavirus genomes and 18 partial gammacoronavirus genomes were retrieved from GenBank. To follow the International Committee on Taxonomy of Viruses (ICTV) *Coronaviridae* demarcation criteria, five conserved domains (3CLpro, NiRAN, RdRp, ZBD, HEL1) were identified via Pfam searches and concatenated (45). Concatenated sequences were aligned using MAFFT v7.520 (46). Maximum likelihood trees were generated using IQ-TREE2 with automatic model selection, 1,000 ultrafast bootstrap replicates, and SH-like test (47). Pairwise distances were calculated to assess intra- and interspecies Pairwise Patristic Distance (PPD) and Pairwise Uncorrected Distance (PUD). Partial RdRp, N, and S genes were analyzed separately using identical methods. Trees were visualized in iTOL with bootstrap ≥70% and SH-aLRT ≥90% considered significant (48). Recombination detection was performed using RDP4 (49), employing seven algorithms (RDP, GENECONV, BootScan, MaxChi, Chimaera, SiScan, and 3Seq), and events detected by three or more methods were considered reliable recombination signals.

## RESULTS

### Bird capture and virus sampling

As part of a large effort to assess zoonotic pathogens and virome in wild bird populations, various species of birds including waterfowl, birds of prey, domestic poultry (chicken and geese), non-waterfowl wild birds, and feral pigeons were captured at different locations in KwaZulu-Natal Province in South Africa (Fig. 1) between February and December in 2018. The pigeons were clinically healthy at the time of capture and sampling. From the captured pigeons, a total of 162 samples were collected which included cloacal (*n* = 81) and oropharyngeal (*n* = 81) swab samples as previously described (33). Samples were stored at −80°C, and the cryovials were placed into a cryogenic container and transported to St. Jude Children’s Research Hospital (Memphis, TN, USA) under cold chain for further analysis.

### Coronavirus identification by PCR

To determine whether the samples contained coronaviruses, RNA was extracted and tested using a pan-coronavirus PCR assay (34). Complementary DNA (cDNA) was synthesized from each RNA sample and subjected to PCR amplification. The PCR products were analyzed via agarose gel electrophoresis to assess the presence of amplified bands, indicating the presence of coronavirus genetic material. Of the 162 pigeon samples, coronavirus RNA was not detected in 154 samples; two additional samples were excluded due to insufficient material.

All the pigeon samples were also tested for IAVs using RT-qPCR and for NDV using conventional RT-PCR as reported previously (33). Eleven (13.6%) cloacal samples, including two from CoV positive samples, obtained from 81 pigeons tested were found positive for IAVs with quantification cycle (Cq) values ≤ 36, whereas none were positive for NDV. Live viruses could not be retrieved from any IAV-positive samples, most likely due to the quality of samples.

### Identification of novel coronaviruses using genome sequencing and genome assembly

In this study, we obtained CoV genome sequences from five different pigeons through enriched library preparation using the Illumina Pan-Coronavirus Panel. All samples yielded sufficient reads to generate draft genomes. Analysis of the CoV sequence reads generated from these samples resulted in one complete CoV genome sequence which was named pigeon gammacoronavirus 18DUR1 (GenBank accession number OR639835) and four near-complete draft CoV genome sequences which were named pigeon gammacoronavirus 18DUR2, 18DUR3, 18DUR4, and 18DUR 5 (GenBank accession numbers OR810020, OR909854, PP236386, and PV863150, respectively). Table 1 shows the characteristics of the five new pigeon gammacoronaviruses reported in the current study including age, sex of pigeons, the date and geolocation of sample collection. All near-complete genomes lacked coverage in parts of Spike, potentially due to the differences in Spike sequences between these coronavirus genome sequences and the sequences used to construct the Illumina enrichment panel.

**TABLE 1:** Characteristics of the five newly discovered gammacoronaviruses reported from feral pigeons in the current study including date and geolocation of sample collection

CoV name	Accession number	Host species	Date of sampling	Age	Sex	Sample material	Location, city, country	Host identifier	Host natural state	Host capture status
18DUR 1	OR639835	Columba livia var. domestica	December, 2018	A^*a*^	U^*b*^	C^*c*^	South Beach, Durban, RSA	Bird 1	Wild (feral)	Active surveillance
18DUR 2	OR810020	Columba livia var. domestica	July, 2018	A	U	C	North Beach, Durban, RSA	Bird 2	Wild (feral)	Active surveillance
18DUR 3	OR909854	Columba livia var. domestica	July, 2018	A	U	C	North Beach, Durban, RSA	Bird 3	Wild (feral)	Active surveillance
18DUR 4	PP236386	Columba livia var. domestica	July, 2018	A	U	C	North Beach, Durban, RSA	Bird 4	Wild (feral)	Active surveillance
18DUR 5	PV863150	Columba livia var. domestica	July, 2018	A	U	OP^*d*^	North Beach, Durban, RSA	Bird 5	Wild (feral)	Active surveillance

^*a*^ A, adult.

^*b*^ U, undetermined.

^*c*^ C, cloacal.

^*d*^ OP, oropharyngeal.

### Genomic features and coding capacity

**Fig 2:**
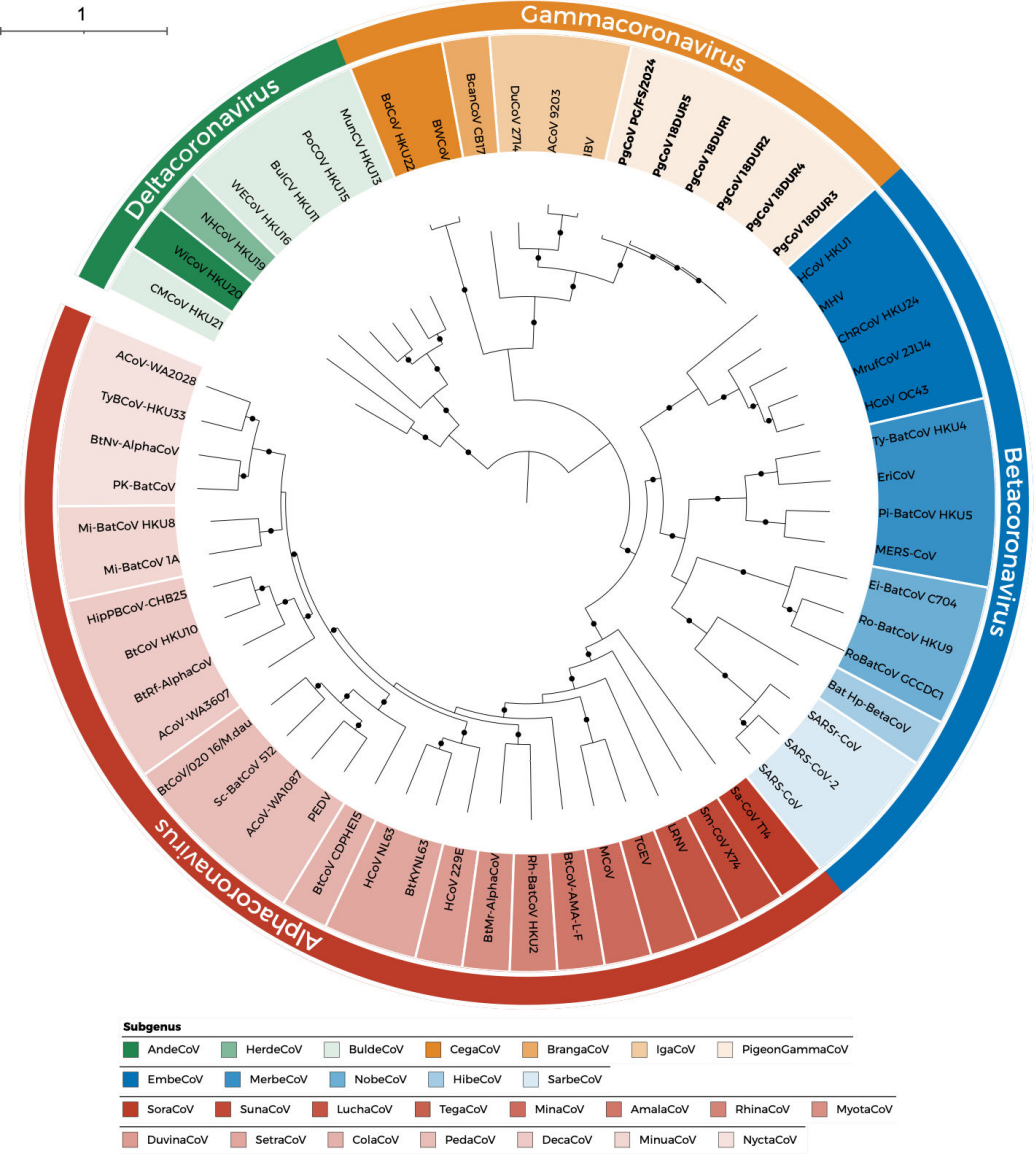
Phylogenetic analysis of coronavirus genomes. Maximum likelihood phylogenetic tree of 58 CoVs constructed from complete genome sequences. The tree was generated using IQ-TREE v2.4.0 with the GTR+F+I+R6 substitution model. Branch support was assessed using 1,000 ultrafast bootstrap replicates and 1,000 SH-aLRT tests. Trees were visualized using iTOL v6. Branches with bootstrap support >70% and SH-aLRT support >90% are indicated by black circles. Pigeon gammacoronaviruses identified in this study are shown in bold. Scale bar indicates nucleotide substitutions per site.

The pigeon gammacoronavirus 18DUR1 (GenBank accession number OR639835) genome was 27,595 bp in length with a G+C content of 37.2%. In the ML phylogeny, the 18DUR1 and other four pigeon gammacoronaviruses formed a distinct clade within the genus Gammacoronavirus (Fig. 2). The genome organization followed the typical of gamma CoVs, with genes arranged in the order 5′-ORF1ab-spike (S)—envelope (E)—membrane (M)—nucleocapsid (N)-3′, flanked by 5′ and 3′ untranslated regions.

**Fig 3:**
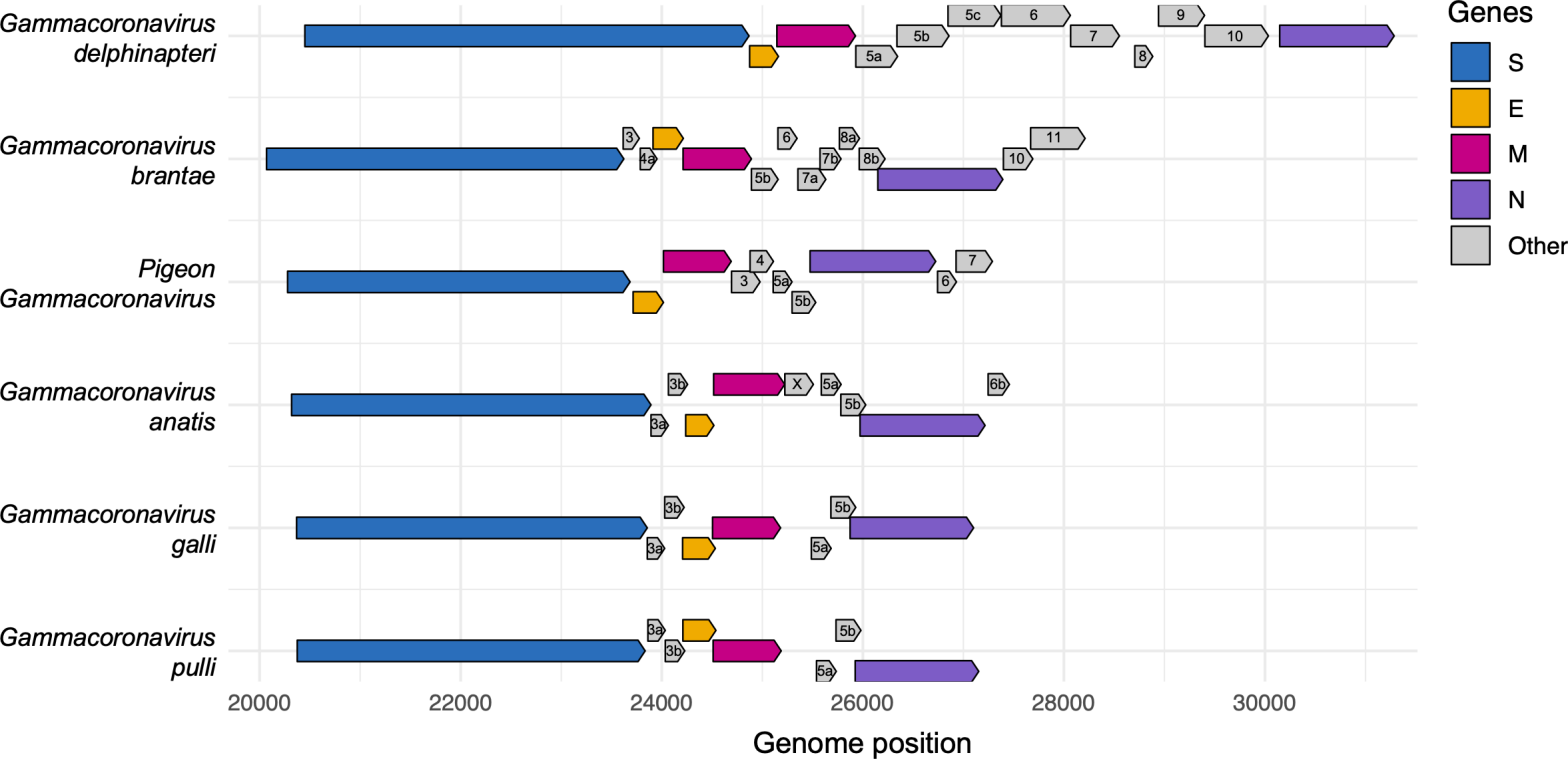
Genome organization of gammacoronaviruses. Schematic representation of genome organization in gammacoronaviruses. Coding sequences (CDSs) downstream of the spike (S) gene are magnified to highlight structural differences between pigeon gammacoronavirus 18DUR1 and six reference gammacoronaviruses. CDSs are displayed vertically according to their respective reading frames.

Unlike other gammacoronaviruses that encode accessory proteins 3a and 3b, pigeon gammacoronavirus did not have these coding sequences (CDSs) but contained predicted accessory genes 3, 4, 5a, 5b, 6, 7 within the genome (Fig. 3). The accessory proteins showed homology to infectious bronchitis virus and other avian gammacoronaviruses, with CDS 3 showing similarity to IBV 4b homolog and CDS 4 to CGGoV 7b homolog, while CDS 6 and 7 showed no significant homology to known coronavirus proteins (Table 2). Non-structural protein (NSP) analysis revealed that ORF1a and ORF1ab encode 16 NSPs with sizes nearly identical to other avian gammacoronaviruses but showing distinct cleavage site patterns (LQ^ST, MQ^VA, and LQ^SS) that differed from *Igacovirus *species (Table 3).

**TABLE 2:** Putative viral proteins of Pigeon gammacoronavirus (18DUR1)

Protein	Top match in NCBI	Query cover (%)	E value	Percent identity	Size (aa)^*d*^
1a	ORF1ab polyprotein [Bird gammacoronavirus CalidrisCN24]	100	0	73.97	3,969
1ab	ORF1ab polyprotein [Bird gammacoronavirus CalidrisCN24]	100	0	81.36	6,660
S	Spike glycoprotein [Bird gammacoronavirus ArenariaCN24]	100	0	73.55	1,136
E	Envelope protein [Bird gammacoronavirus AnasCN24]	99	7e−59	89	101
M	Membrane protein [Bird gammacoronavirus AnasCN24]	100	1e−151	90.67	225
3^*a*^	Hypothetical protein [Bird gammacoronavirus LimosaCN24]	96	2e−34	64.84	94
4^*b*^	Hypothetical protein [Bird gammacoronavirus CharadriusCN24]	99	1e−24	63.29	77
5a	5a protein [Bird gammacoronavirus TringaCN24]	97	4e−23	72.13	63
5b	5b protein [Bird gammacoronavirus CharadriusCN24]	99	4e−35	76.92	79
N	Nucleocapsid protein [Bird gammacoronavirus CalidrisCN24]	100	0	82.78	417
6	n/a^*c*^				63
7	Hypothetical protein [Feral pigeon parvovirus A]	80	1e−10	38.24	121

^*a*^ IBV 4b homolog.

^*b*^ Canada Goose Coronavirus (CGCoV) 7b homolog.

^*c*^ n/a, no significant BLAST hit found.

^*d*^ aa, amino acids.

**TABLE 3:** Non-structural proteins size and cleavage site of gammacoronaviruses

Protein	*delphinapteri*		*brantae*		*columbae*		*anatis*		*galli*		*pulli*	
	Cleavage site	Size (aa)^*c*^	Cleavage site	Size (aa)	Cleavage site	Size (aa)	Cleavage site	Size (aa)	Cleavage site	Size (aa)	Cleavage site	Size (aa)
NSP1/2	VD^GD^*a*^	636	AG^GH	609	AG^GK	670	AG^GK	670	AG^GK	673	AG^GK	673
NSP3	LG^GV	1,586	AG^GV	1,532	AG^GV	1,610	AG^GV	1,623	AG^GI	1,592	AG^GV	1,594
NSP4	LQ^AG	537	LQ^AG	503	LQ^AG	513	LQ^AG	515	LQ^SG	514	LQ^TG	514
NSP5	LQ^SN	303	LQ^SN	307	LQ^ST	307	LQ^SS	307	LQ^SS	307	LQ^SS	307
NSP6	VQ^SK	303	VQ^SK	295	VQ^SK	295	VQ^SK	294	VQ^AK	293	VQ^SK	293
NSP7	LQ^AV	83	LQ^AV	83	LQ^SV	83	LQ^SV	83	LQ^SV	83	LQ^SV	83
NSP8	LQ^NN	198	LQ^NN	212	LQ^NN	210	LQ^NN	210	LQ^NN	210	LQ^NN	210
NSP9	LQ^HG	112	LQ^GK	111	LQ^SK	111	LQ^SK	111	LQ^SK	111	LQ^SK	111
NSP10	LQ^SV	189	–	173	MQ^VA	143	VQ^SD	145	VQ^SV	145	VQ^SV	145
NSP11	–	17	–	–	–	27	–	22	–	23	–	23
NSP12	LQ^AS	926	LQ^SC	1,101	LQ^SC	959	LQ^SC	956	LQ^SC	940	LQ^SC	940
NSP13	LQ^SQ	601	LQ^SN	599	LQ^ST	600	LQ^ST	600	LQ^GT	600	LQ^GT	600
NSP14	LQ^SL	528	LQ^SI	522	LQ^SI	521	LQ^SI	522	LQ^SI	514	LQ^SI	521
NSP15	LQ^SD	349	LQ^SG	338	LQ^SS	338	LQ^SA	338	LQ^SA	338	LQ^SA	338
NSP16		312	–^*b*^	298	–	278	–	277	–	302	–	302

^*a*^ “^” represents the cleavage site of a protease.

^*b*^ “–” denotes the corresponding nsp is not present.

^*c*^ aa, amino acids.

### Phylogenetic analysis of pigeon gammacoronavirus

**Fig 4:**
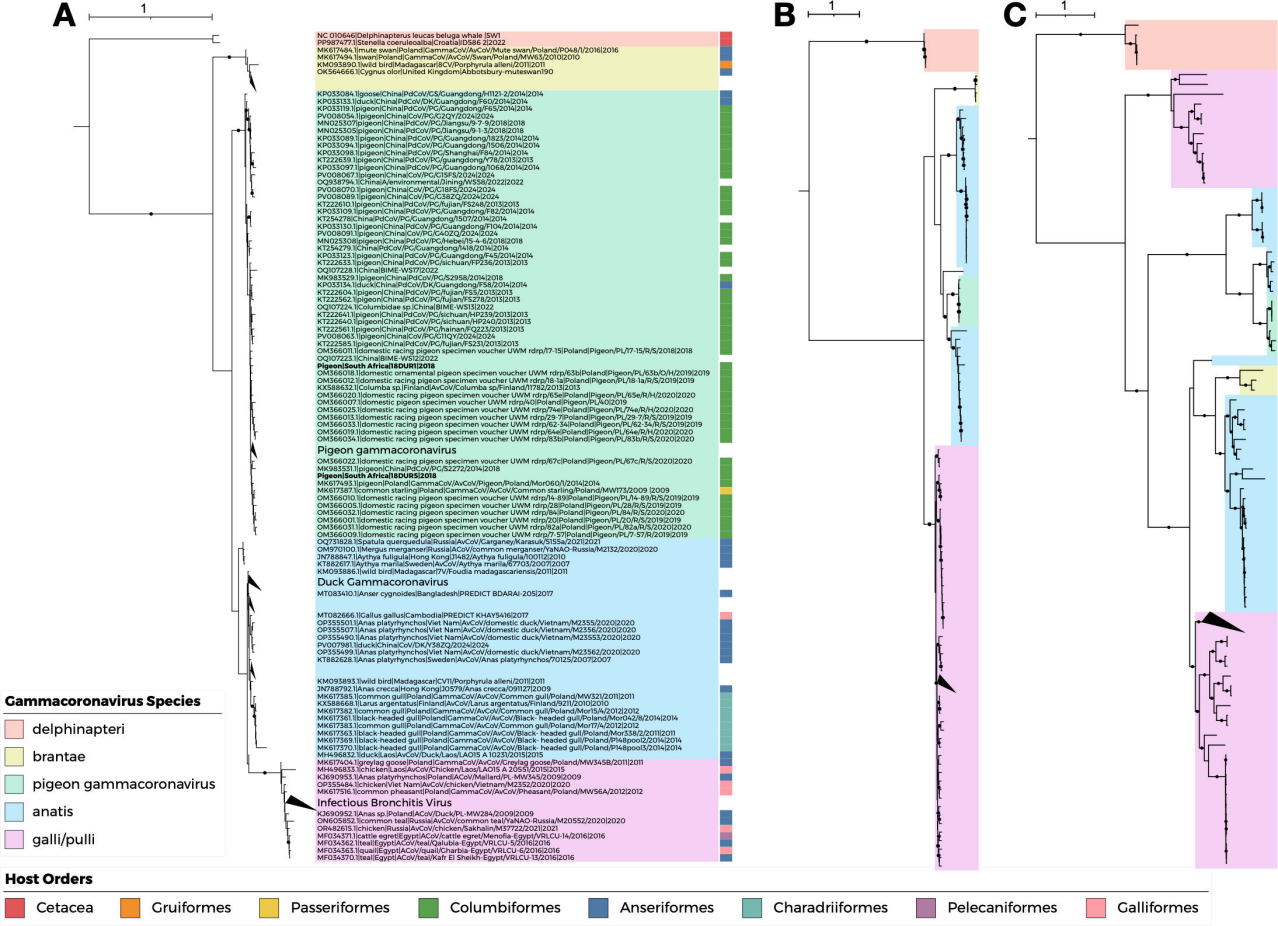
Phylogenetic analysis of gammacoronavirus genes. (**A**) Maximum likelihood (ML) tree of 352 gammacoronaviruses constructed from partial RdRp gene sequences. The tree was generated using IQ-TREE v2.4.0 with the TIM3+F+R4 substitution model. Branch support was assessed using 1,000 ultrafast bootstrap replicates and 1,000 SH-aLRT tests. Trees were visualized using iTOL v6. Branches with bootstrap support >70% and SH-aLRT support >90% are indicated by black circles. Identical pigeon gammacoronavirus genome sequences were removed, and CoV sequences identified in this study are shown in bold. Scale bar indicates nucleotide substitutions per site. (**B**) ML tree of 327 gammacoronaviruses constructed from nucleocapsid gene sequences. Tree construction and visualization parameters are as described in panel A. (**C**) ML tree of 331 gammacoronaviruses constructed from spike gene sequences. The tree was generated using IQ-TREE v2.4.0 with the GTR+F+I+R7 substitution model. Other parameters are as described in panel A.

ML phylogenetic analysis of the partial RdRp gene (382 bp) showed that the 18DUR1 and 18DUR5 formed a distinct cluster with previously reported pigeon gammacoronavirus although these two South African gammacoronavirus sequences clustered in different clades within the pigeon gammacoronavirus group (Fig. 4A).

Pigeon gammacoronavirus was also detected in *Anseriformes *and *Passeriformes *(common starling) based on partial RdRp sequences. Phylogenetic analyses of nucleocapsid and spike genes showed closer relationships with duck coronavirus, with gene-specific variations particularly evident in spike gene phylogeny (Fig. 4B and C). Recombination detection using RDP4 across seven algorithms consistently identified a fragment of the S gene of 18DUR1 as recombinant with 18DUR4 and 18DUR5, providing evidence of intra‐regional recombination among pigeon gammacoronaviruses (Fig. S1).

### Gammacoronavirus species demarcation

**Fig 5:**
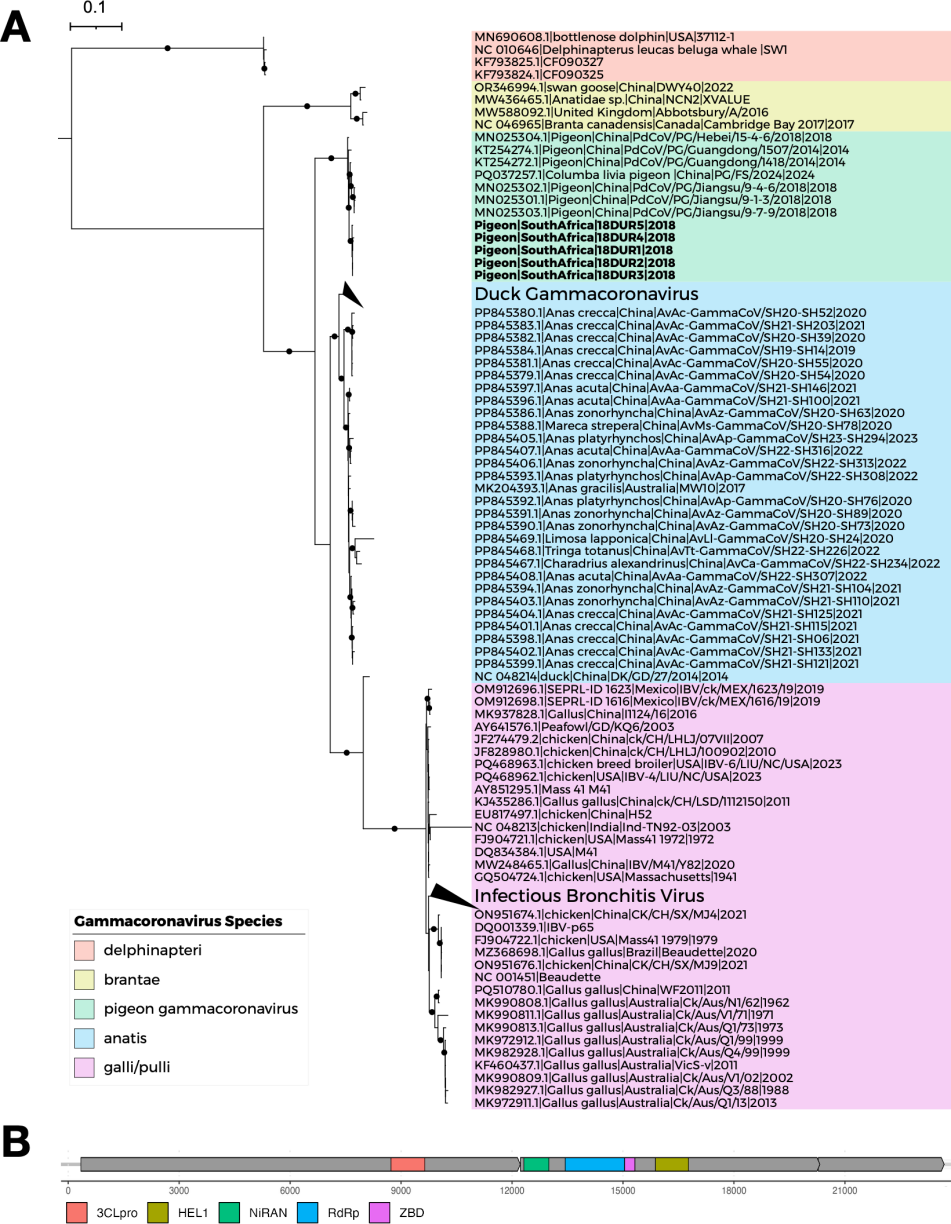
Phylogenetic analysis of five conserved protein domains. (**A**) Maximum likelihood (ML) phylogenetic tree of 759 gammacoronaviruses constructed from concatenated sequences of five conserved domains. The tree was generated using IQ-TREE v2.4.0 with the Q.mammal+F+I+R6 substitution model. Branch support was assessed using 1,000 ultrafast bootstrap replicates and 1,000 SH-aLRT tests. Trees were visualized using iTOL v6. Branches with bootstrap support >70% and SH-aLRT support >90% are indicated by black circles. Identical gammacoronavirus sequences were removed, and pigeon gammacoronavirus genome sequences identified in this study are shown in bold. Scale bar indicates amino acid substitutions per site. (**B**) Schematic representation of the five conserved protein domains used for phylogenetic analysis.

**Fig 6:**
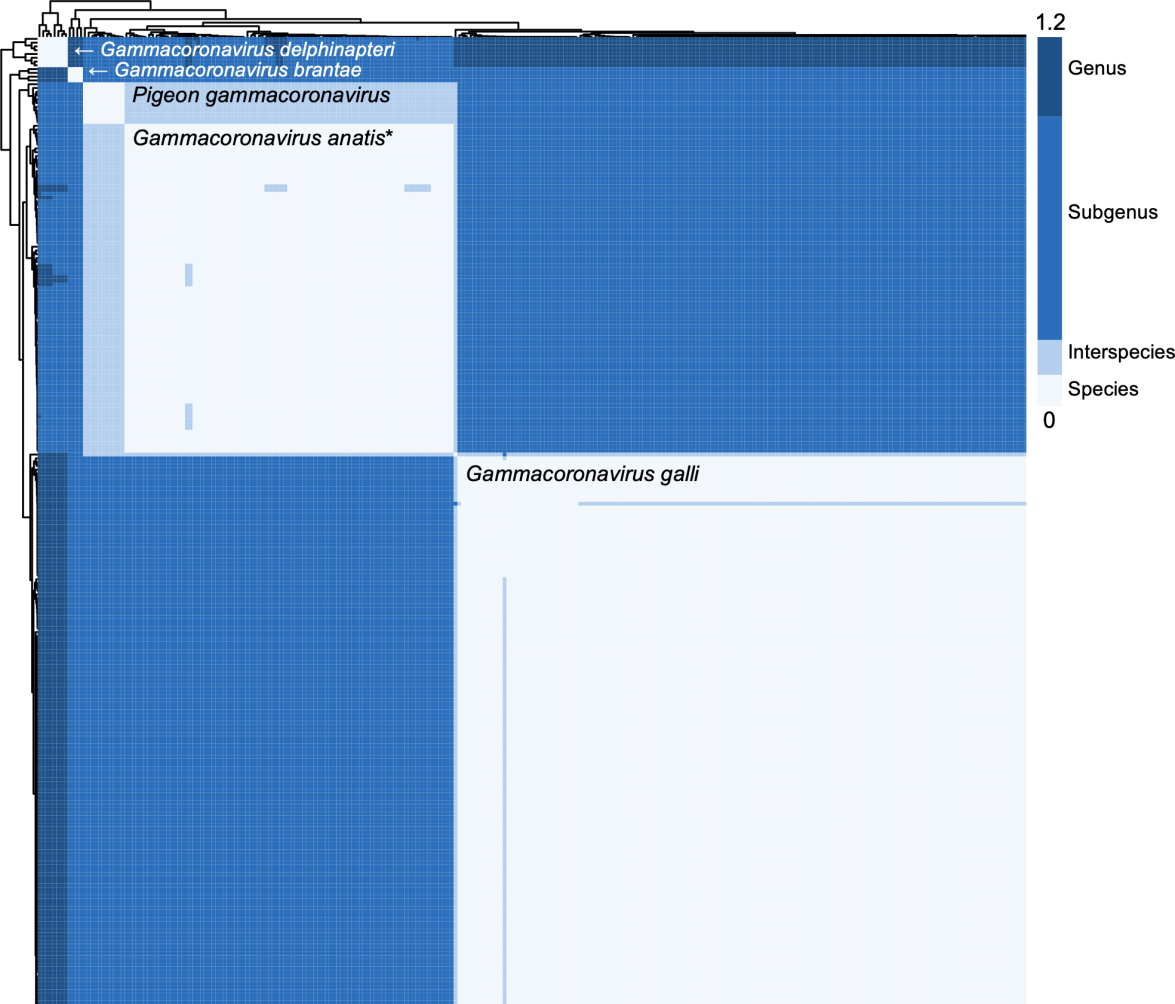
Species demarcation matrix of gammacoronaviruses with phylogenetic tree. Species demarcation matrix based on pairwise patristic distance (PPD) values calculated from concatenated sequences of five conserved protein domains of 931 gammacoronaviruses. The corresponding maximum likelihood phylogenetic tree is shown adjacent to the matrix. Color coding indicates species demarcation categories based on Coronaviridae taxonomic thresholds. The matrix display was cropped due to the large number of IBV genome sequences included in the analysis. The “*“ denotes GenBank accession number PP845379 for the newly identified *Gammacoronavirus anatis*.

According to ICTV *Nidovirales *demarcation criteria, phylogenetic analysis of concatenated conserved domains (3CLpro, NiRAN, RdRp, ZBD, HEL1) showed that pigeon gammacoronavirus formed a distinct clade with previously reported Chinese pigeon gammacoronavirus, clustering closely with duck coronavirus (Fig. 5). PPD and PUD analyses demonstrated that pigeon gammacoronavirus represents a novel species, as the calculated PPD values surpassed the ICTV cutoffs for coronavirus species and subgenus demarcation. Accordingly, it clusters with the duck coronavirus (*Gammacoronavirus anatis*) (accession number PP845379) whcih was recently reported by Xu et al. (50) to form a distinct new subgenus (Table 4; Fig. 6; Fig. S2). The newly identified *Gammacoronavirus anatis* (accession number PP845379) formed a separate clade from the previously classified *Gammacoronavirus anatis * (accession number NC_048214), indicating that duck gammacoronaviruses should be divided into two distinct species.

**TABLE 4:** Pairwise patristic distances (PPD) and pairwise uncorrected distances (PUD) of gammacoronavirus species based on concatenated five domains (3CLpro, NiRAN, RdRp, ZBD, and HEL1)

Species	Number of sequences		Intraspecies				Interspecies			
	Intra species	Inter species	PPD		PUD		PPD		PUD	
			Mean	Range	Mean	Range	Mean	Range	Mean	Range
*Gammacoronavirus delphinapteri*	8	927	0.00	0.00–0.01	0.00	0.00–0.01	1.08	0.89–1.17	0.30	0.29–0.34
*Gammacoronavirus brantae*	4	931	0.04	0.01–0.06	0.03	0.01–0.05	0.54	0.34–0.94	0.21	0.17–0.31
*Pigeon Gammacoronavirus*	12	923	0.01	0.00–0.02	0.01	0.00–0.02	0.33	0.13–0.92	0.14	0.08–0.30
*Gammacoronavirus anatis* ^*a*^	1	934	–^*c*^				0.19	0.11–0.94	0.10	0.07–0.30
*Gammacoronavirus anatis*^*b*^	87	848	0.04	0.00–0.11	0.03	0.00–0.07	0.29	0.11–0.95	0.15	0.07–0.30
*Gammacoronavirus pulli*	1	934	–				0.17	0.08–1.08	0.10	0.06–0.34
*Gammacoronavirus galli*	823	112	0.06	0.00–0.18	0.03	0.00–0.12	0.36	0.14–1.17	0.16	0.08–0.34

^*a*^ The previously classified *Gammacoronavirus anatis * (accession number NC_048214).

^*b*^ The newly identified *Gammacoronavirus anatis* (accession number PP845379).

^*c*^ “–” denotes intraspecies distance not applicable (single sequence available).

Collectively, the phylogenetic and sequence analyses suggest that the five pigeon CoVs identified in this study represent a genetically distinct lineage within the *Gammacoronavirus* genus. These findings highlight the underappreciated diversity of gammacoronaviruses in avian species and emphasize the importance of continued genomic surveillance to better understand their evolution and potential host range.

## DISCUSSION

The discovery of novel gammacoronaviruses in wildlife populations continues to reveal the remarkable genetic diversity within the *Coronaviridae* family. Here, we report the identification and genomic characterization of five pigeon gammacoronaviruses in feral pigeons captured in South Africa. These viruses represent a novel species *Gammacoronavirus columbae* that expands our understanding of coronavirus diversity in avian hosts.

Coronavirus infections in their natural hosts, including avian and mammalian species, can be asymptomatic, contributing to their efficient maintenance and spread in wildlife populations. The CoV-infected pigeons in the present study were clinically healthy at the time of sampling, which may be explained by the replication of CoVs in non-respiratory enteric epithelial cells which normally does not result in clinical disease (10, 22). Fecal excretion of the virus may shed CoVs at sufficient doses in fecal materials or contaminated fomites to infect other susceptible hosts. The detection of gammacoronaviruses in clinically healthy pigeons suggests that these birds may serve as a natural reservoir species that may facilitate viral persistence and transmission without apparent disease, mirroring similar asymptomatic infections seen in other coronavirus-host systems. It remains unclear whether feral pigeons serve as active hosts for gammacoronaviruses or merely act as mechanical vectors. The detection of CoV genomes in cloacal swabs in the current study indicates possible viral shedding through fecal excretion, yet the absence of clinical disease and 6.2% prevalence observed suggests these viruses have evolved stable host-pathogen relationships that favor viral persistence over acute pathogenesis.

This study establishes pigeon gammacoronavirus as a novel species within the *Gammacoronavirus* genus and supports the creation of a new subgenus to accommodate emerging avian coronavirus diversity. Phylogenetic analysis using concatenated conserved domains (3CLpro, NiRAN, RdRp, ZBD, HEL1) (51, 52) demonstrates that pigeon gammacoronavirus forms a distinct clade with previously reported Chinese pigeon gammacoronaviruses, while clustering closely with novel duck coronavirus (50). Genetic distance analyses support species-level classification, with pigeon gammacoronavirus exhibiting a narrow intraspecies PPD range (0.00–0.02) compared to substantial interspecies divergence (0.13–0.92).

Additionally, duck gammacoronaviruses comprise two distinct species rather than a single taxonomic unit. The recently reported *Gammacoronavirus anatis* (accession number PP845379) forms a separate clade from the previously classified *Gammacoronavirus anatis* (accession number NC_048214), indicating that diversity within duck-associated gammacoronaviruses has been underestimated. Pigeon gammacoronavirus and *Gammacoronavirus anatis* (PP845379) clustered together to form a new subgenus within Gammacoronavirus, indicating that the current taxonomic frameworks require revision to reflect avian coronavirus diversity.

The genome organization of pigeon gammacoronavirus followed the conserved structure of other gammacoronaviruses, yet exhibits distinctive features that reflect evolutionary divergence within the genus. Unlike Brangacovirus and Igacovirus, which have accessory proteins between S-E regions, this pigeon gammacoronavirus lacks accessory proteins between S and E, similar to Cegacovirus. The absence of typical gammacoronavirus accessory proteins 3a and 3b, coupled with the presence of novel CDSs 6 and 7 lacking homology to known coronavirus proteins, suggests functional divergence that may reflect adaptive responses to the pigeon host environment. The presence of IBV 4b and CGCoV 7b homologs (CDSs 3 and 4, respectively) indicates selective preservation of certain ancestral functions, while the evolution of novel accessory proteins may confer pigeon-specific advantages in viral fitness or immune evasion. The distinct cleavage site patterns observed in non-structural proteins (LQ^ST, MQ^VA, LQ^SS) further differentiate pigeon gammacoronavirus from other *Igacovirus* species.

The substantial divergence observed in the Spike protein compared to other viral proteins is particularly significant given its role in receptor binding and host cell entry (53). Phylogenetic analyses of the spike gene showed closer relationships with duck coronavirus, suggesting distinct evolutionary pressures on host-virus interaction mechanisms and adaptation to pigeon-specific cellular receptors, while the conservation of other genomic regions reflects functional constraints on essential viral processes. Notably, targeted enrichment sequencing revealed gaps in Spike gene coverage, likely reflecting sequence divergence from panel reference sequences and highlighting the rapid evolution of S protein.

The detection of pigeon gammacoronavirus in *Anseriformes *and *Passeriformes *based on partial RdRp sequences suggests a broader host range than initially anticipated though the limitations of partial sequences preclude definitive determination and the detection of gammacoronaviruses in clinically healthy pigeons suggests that these birds may act as natural reservoirs, facilitating viral persistence and transmission without apparent disease (32). The formation of distinct clades by Charadriiformes within duck coronavirus phylogenies suggests that gammacoronavirus diversity extends beyond simple host-order associations. These findings suggest that the true diversity of gammacoronaviruses in wild birds may be far greater than currently described, particularly as sampling expands to under-sampled taxa such as Passeriformes.

Next-generation sequencing is likely to accelerate virus discovery compared to conventional approaches. As the costs of NGS continue to decline, obtaining complete or draft viral genome sequences has become increasingly feasible, enabling simultaneous detection of both known and novel viral species without requiring virus isolation (54–56).

The identification of these viruses in South African feral pigeons expands our geographic understanding of gammacoronavirus distribution in wild bird populations. The 6.2% prevalence observed suggests these viruses may be more widespread than previously recognized. The emergence of SARS-CoV-2 has underscored the importance of understanding ecological distributions of coronavirus in animal reservoirs. While the genetic divergence observed suggests limited immediate risk for human infection, the potential for spillover to poultry remains a concern given the close phylogenetic relationship between pigeon and duck coronaviruses. Additional studies are needed to assess viral load, isolate live virus, and conduct pathogenesis and persistence experiments to determine whether pigeons serve as natural reservoirs for coronaviruses with potential public health implications, as transmission events could have implications for domestic poultry health and warrant inclusion of pigeon in surveillance programs, paralleling similar efforts in bats (53).

This study enhances our understanding of CoV ecology, diversity, and geographical distribution while emphasizing the need for large-scale surveillance efforts crucial for CoV disease control and prevention in susceptible hosts. Our findings highlight the essential role of NGS in genomic surveillance for detecting both divergent and novel CoVs. Although the diversity of coronaviruses in wild birds remains underappreciated, NGS has proven invaluable in zoonotic surveillance, enabling the discovery of novel viral sequences in avian populations (57–59). In this study, we identified pigeon gammacoronaviruses that diverge significantly from existing species. With expanding feral pigeon populations due to urbanization and new settlements, systematic CoV monitoring—including surveillance, characterization, diagnosis, and disease management—is increasingly critical for both human and animal health. Although this study does not directly assess the potential transmission of avian CoVs to humans or other animals, the risk of spillover to non-human hosts, particularly poultry, remains a concern. Our findings contribute to a deeper understanding of CoV diversity in wild birds and underscore the need for vigilance in monitoring emerging CoVs to mitigate future spillover risks. In summary, the discovery of pigeon gammacoronavirus 18DUR1-18DUR5 expands our understanding of avian gammacoronavirus ecology. Given its potential for cross-species transmission, future research should focus on host tropism, viral shedding, and possible recombination events that could impact pathogenicity.

## Data Availability

The nucleotide sequence data of the five gammacoronavirus (18DUR1, 18DUR2, 18DUR3, 18DUR4, and 18DUR5) genomes that support the findings of the present study have been deposited in the GenBank database of the National Center for Biotechnology Information under BioProject number PRJNA912654 and under nucleotide sequence accession numbers OR639835, OR810020, OR909854, PP236386, and PV863150, respectively.

## SUPPLEMENTAL MATERIAL

Figures S1 to S6 jvi.01112-25-s0001.pdf Recombination analysis, pairwise patristic distance distribution, and expanded phylogenetic trees of pigeon gammacoronaviruses.

ASM does not own the copyrights to Supplemental Material that may be linked to, or accessed through, an article. The authors have granted ASM a non-exclusive, world-wide license to publish the Supplemental Material files. Please contact the corresponding author directly for reuse.
