# Supplementary material for: Discovery, phylogenetic, and comparative genomic analysis of novel avian gammacoronaviruses identified in feral pigeons (Columba livia domestica)
Source: J Virol. 2025 Aug 20;99(9):e01112-25. doi: 10.1128/jvi.01112-25 (PMC12456012; doi:10.1128/jvi.01112-25)

Fig. S1. Recombination analysis of pigeon gammacoronaviruses. Results of recombination analysis performed on five pigeon gammacoronavirus genomes identified in this study using RDP4. Sequence alignments were gap-stripped using Geneious prior to analysis. Window and step size parameters were set to default values.

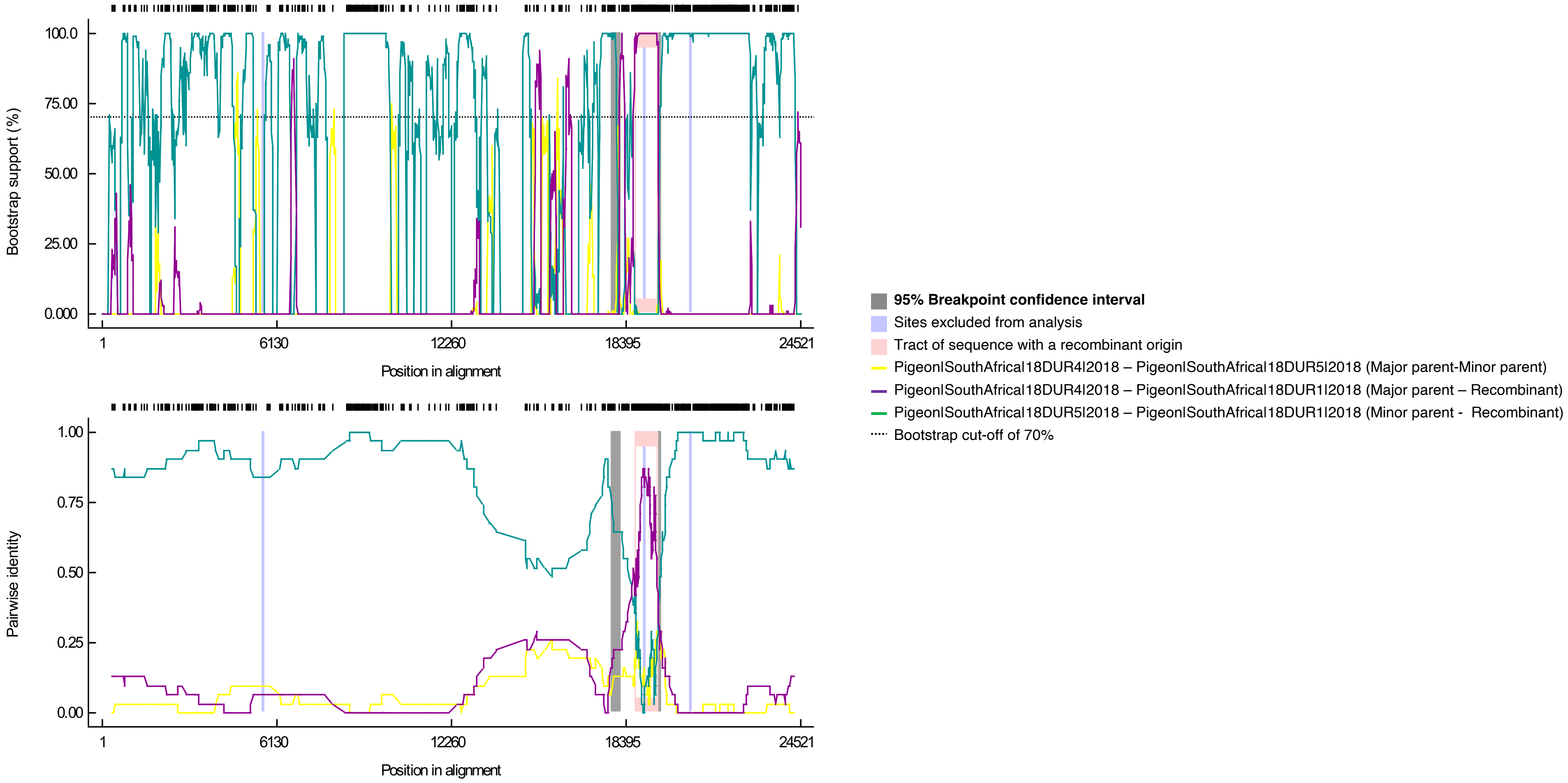

Fig. S2. Pairwise patristic distance distribution of gammacoronaviruses. Ridge plot displaying the distribution of pairwise patristic distance (PPD) values calculated from concatenated sequences of five conserved protein domains of 935 gammacoronaviruses.

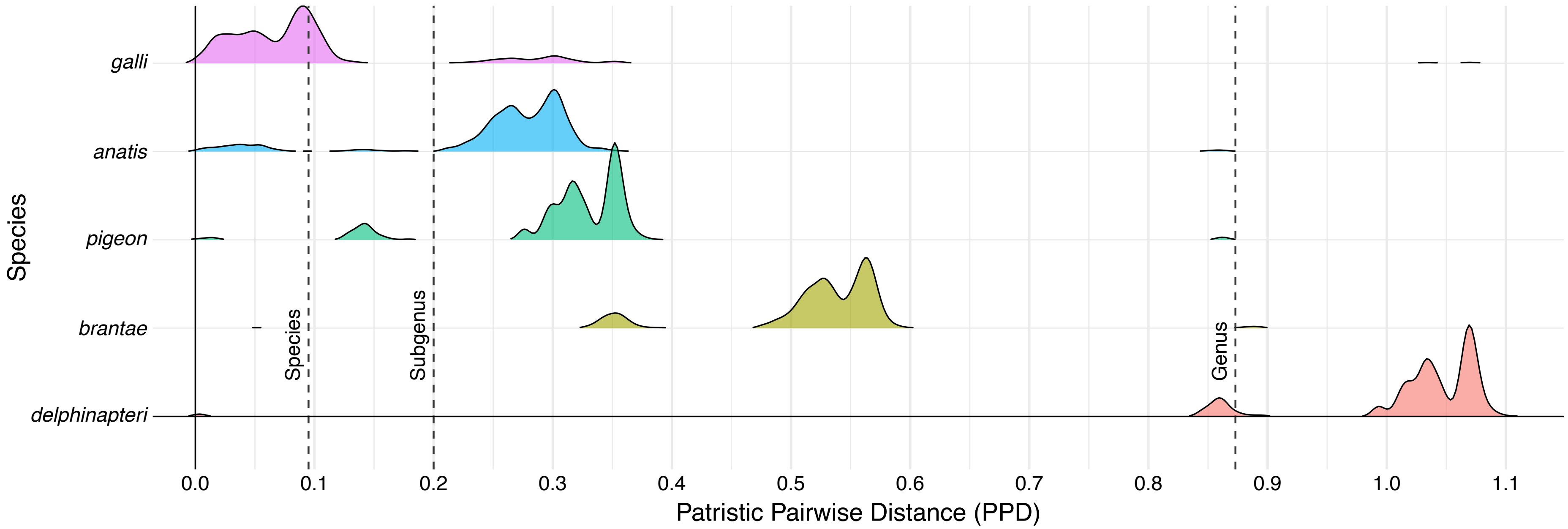

Fig. S3. Expanded phylogenetic tree from Figure 4A. Complete phylogenetic tree with all taxa visible, corresponding to the collapsed version presented in Figure 4A.

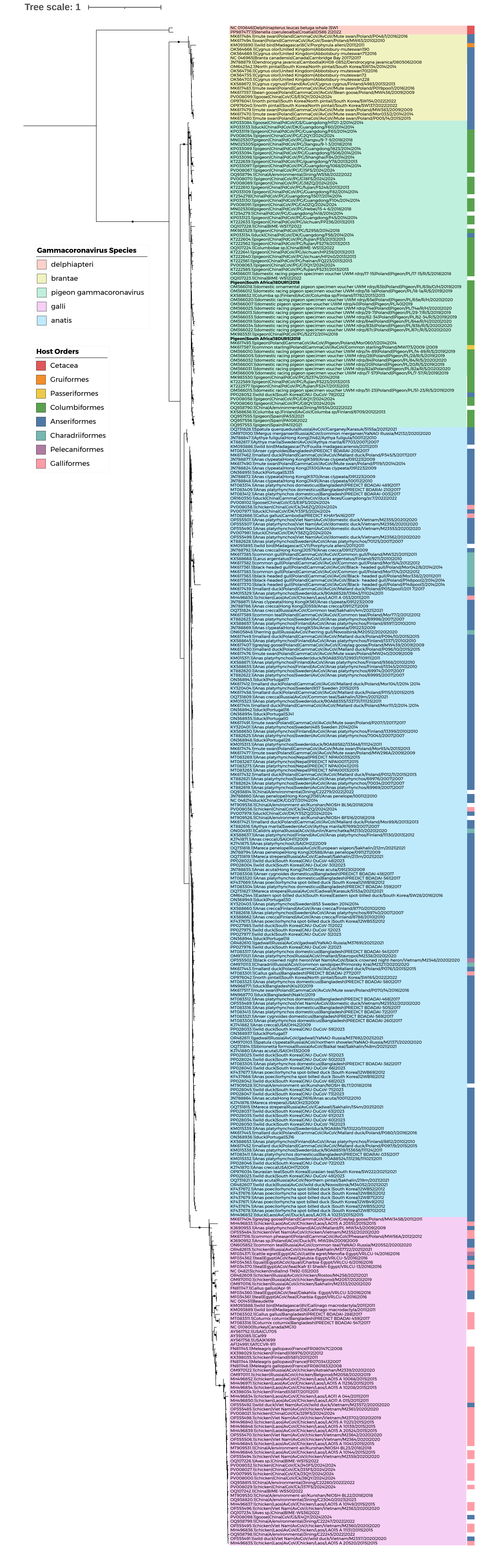

Fig. S4. Expanded phylogenetic tree from Figure 4B. Complete phylogenetic tree with all taxa visible, corresponding to the collapsed version presented in Figure 4B.

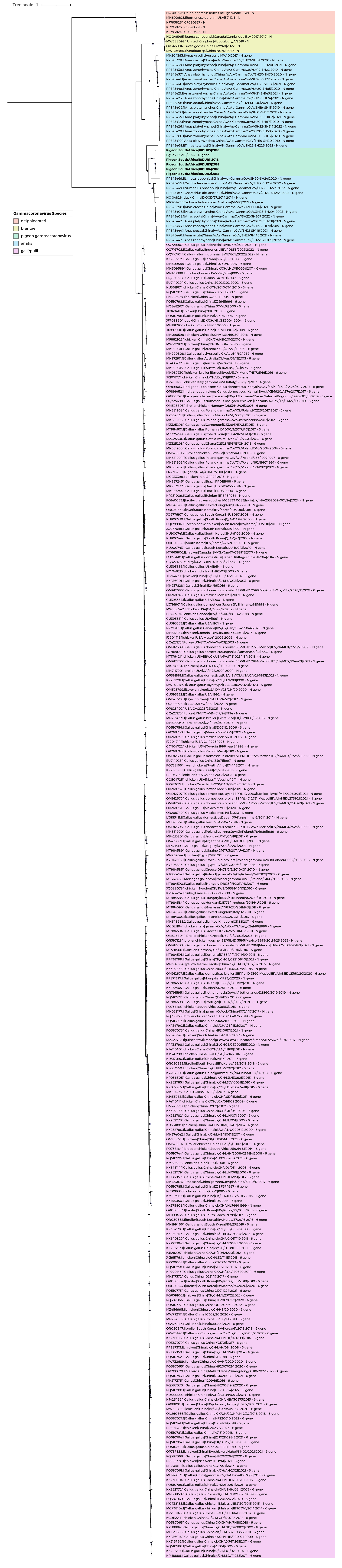

Fig. S5. Expanded phylogenetic tree from Figure 4C. Complete phylogenetic tree with all taxa visible, corresponding to the collapsed version presented in Figure 4C.

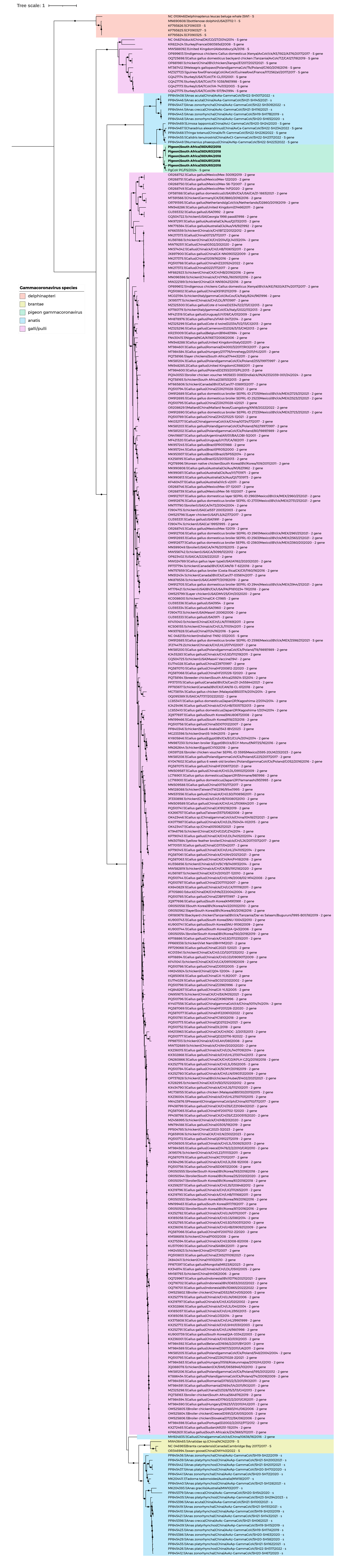

Fig. S6. Expanded phylogenetic tree from Figure 5. Complete phylogenetic tree with all taxa visible, corresponding to the collapsed version presented in Figure 5.

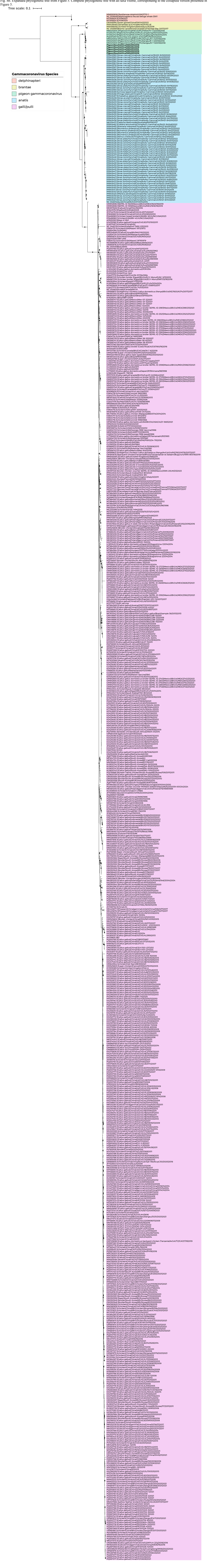

Supplement: Figures S1 to S6 — Recombination analysis, pairwise patristic distance distribution, and expanded phylogenetic trees of pigeon gammacoronaviruses. [file jvi.01112-25-s0001.pdf]
